# A rare case of a patient with PPP syndrome presenting pancreatic pseudocysts, panniculitis, and symptoms of polyarthritis. A radicular cyst of the upper jaw could be another manifestation of the syndrome

**DOI:** 10.1002/ccr3.2651

**Published:** 2020-01-14

**Authors:** Wolfgang Konschake, Thea Westphal, Michael Jünger, Andreas Arnold, Stine Lutze

**Affiliations:** ^1^ Department of Dermatology University of Greifswald Greifswald Germany

**Keywords:** ghost cells, pancreatitis, panniculitis, polyarthritis, PPP syndrome, radicular cyst

## Abstract

In rare cases, pancreatic enzymes can enter the bloodstream and cause fat necrosis in the bone and tissue leading to a disorder called pancreatitis, panniculitis, and polyarthritis syndrome. Clinicians should have this syndrome in mind when treating patients with pancreatitis.

## Graphical abstract

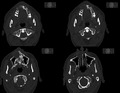

## 1 CASE REPORT

We report a case of a patient suffering from pancreatitis, panniculitis, and polyarthritis (PPP) syndrome, an extremely rare disease resulting from pancreatic fat necrosis in the bone and dermal tissue. In addition, we discuss a radiculodental cyst in the maxilla with a causal relationship to the syndrome.

**Figure 1:**
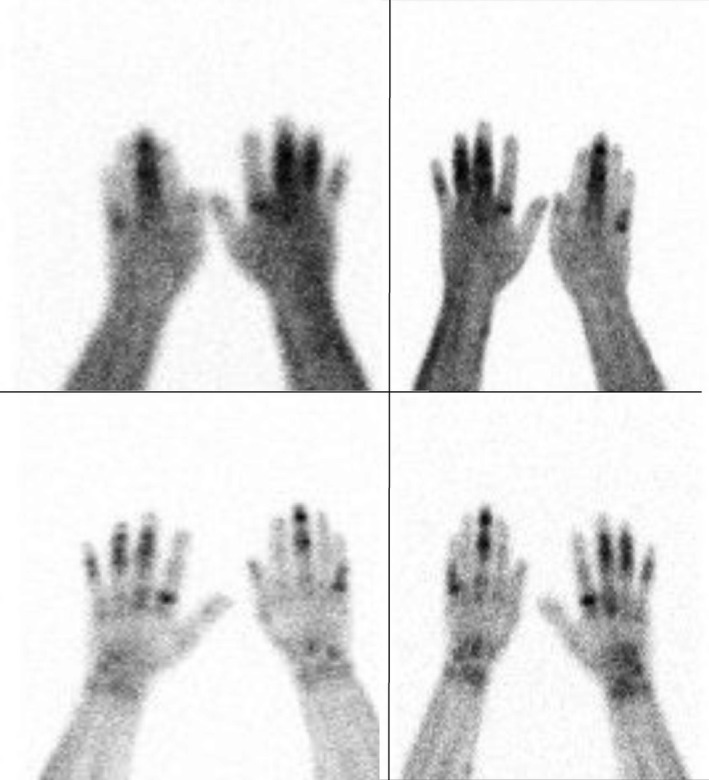
Active bone processes (ostitis/osteomyelitis) in digitus II, III, IV, and V right and in left and right digit III

**Figure 2:**
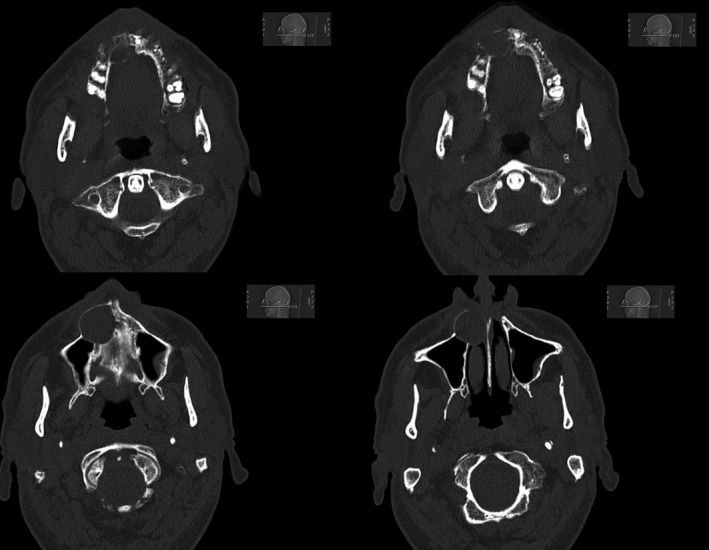
CT midface shows a radico dental cyst in the upper jaw right

We saw a fairly young Caucasian male patient for diagnosis and further treatment of nodular skin changes on the legs. Despite a chronic nicotine dependence with sclerosis of the aorta, the patient did not exhibit any comorbid conditions or use of long‐term medication. A slight injury on the left middle finger due to forestry work led to the admission to the Department of Traumatology. The initial diagnosis resulted in a phlegmon of the extensor tendons with reactive osteomyelitis of different fingers on both hands (Figure 1). The patient was, therefore, treated for a surgical wound and incision debridement on the afflicted fingers. Furthermore, an antibiotic therapy with clindamycin was initiated. The patient suffered from pronounced toothache during hospitalization. Colleagues in oral and maxillofacial surgery treated this by tooth extraction and radiculodental cyst resection in the right upper jaw (Figure 2).

**Figure 3:**
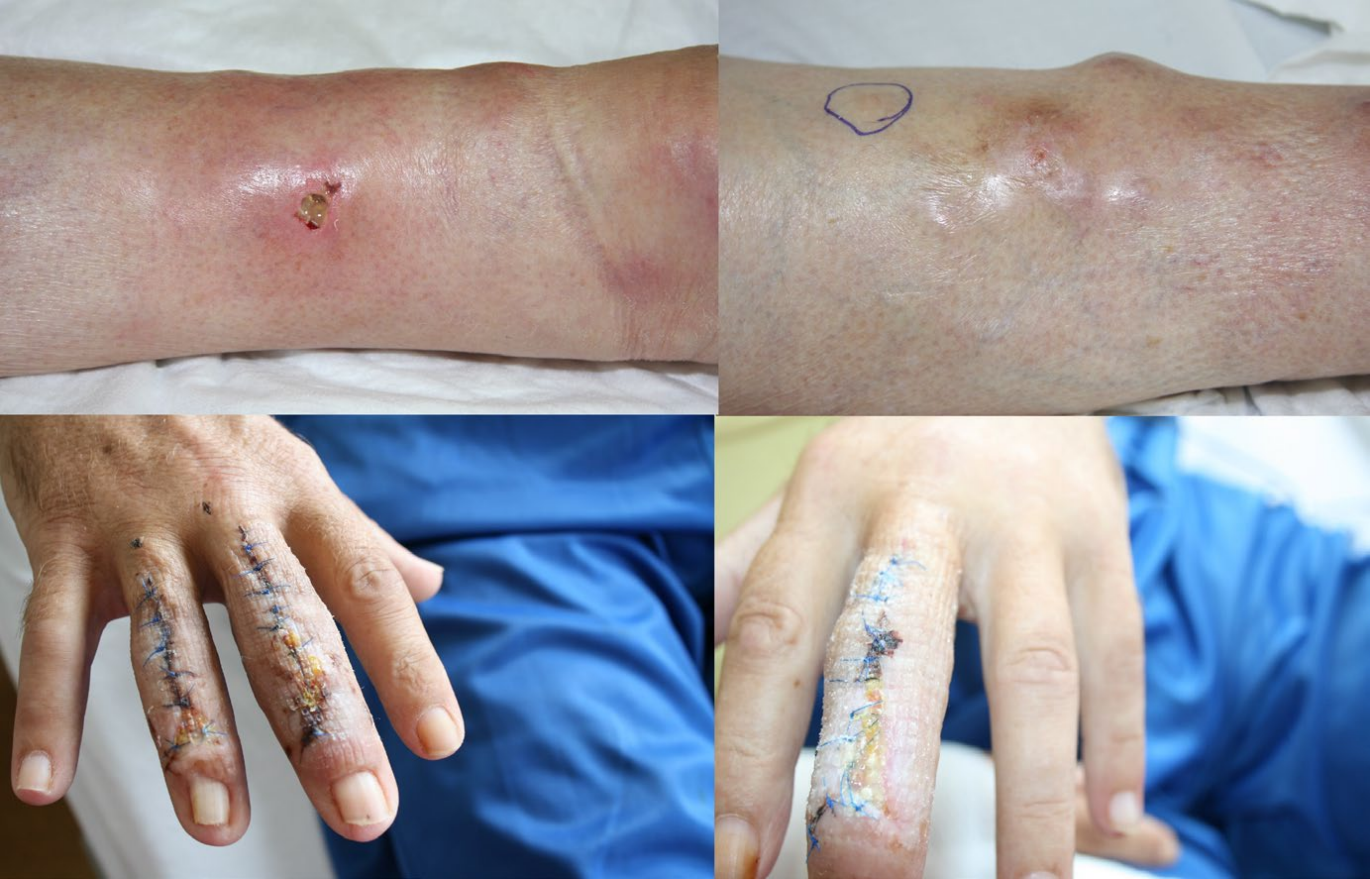
Disseminated subcutaneous dermal infiltrated, livid discolored nodules on lower legs. Swollen, reddened fingers with yellowish fibrinous occupied ulcers

**Figure 4:**
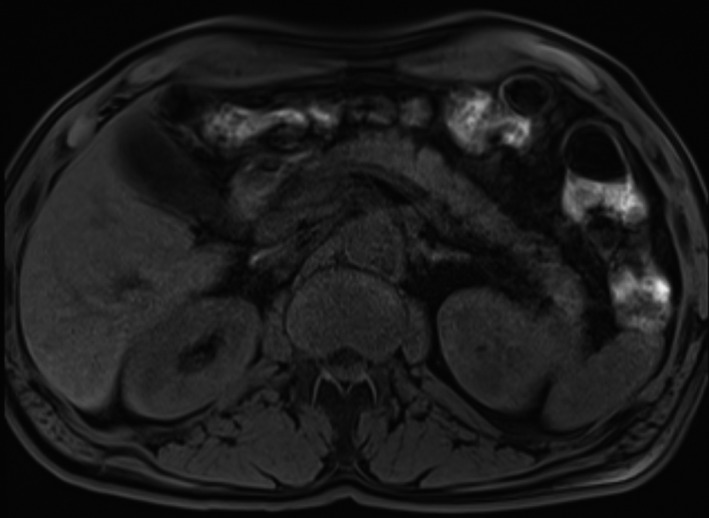
Cystic imposing, about 2 × 4 cm lesion in the area of the pancreatic tail

**Figure 5:**
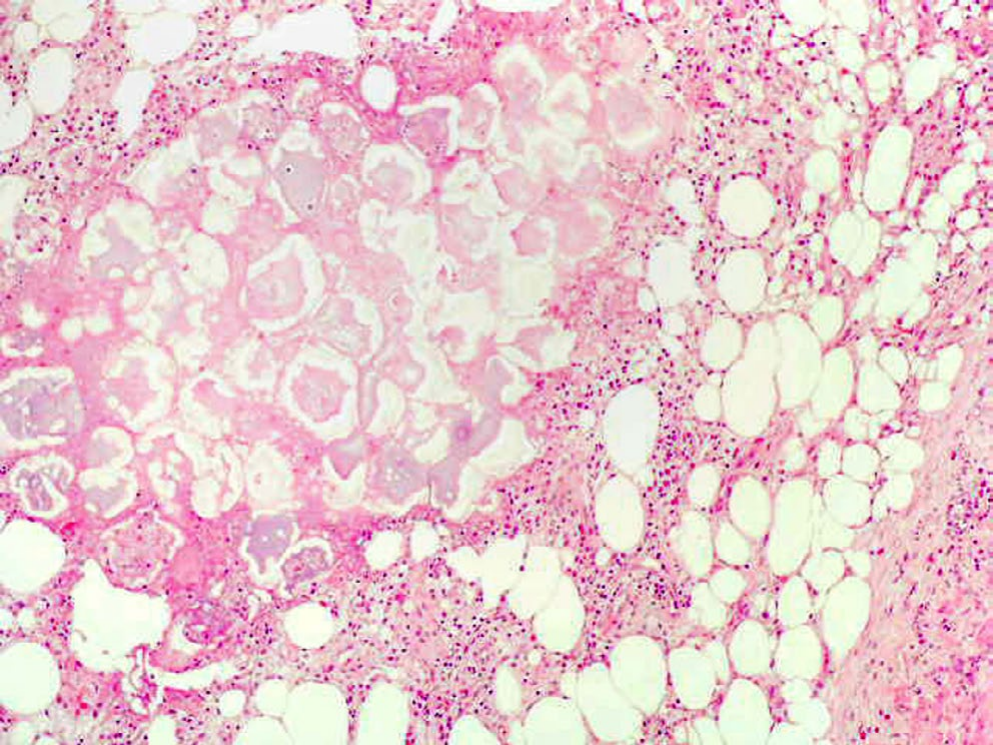
Histology of a biopsy taken from a nodus on the right leg. Adipocytes in the center lost there their nucleiius and show a thickened membrane leading to the image of ghost‐like adipocytes

**Figure 6:**
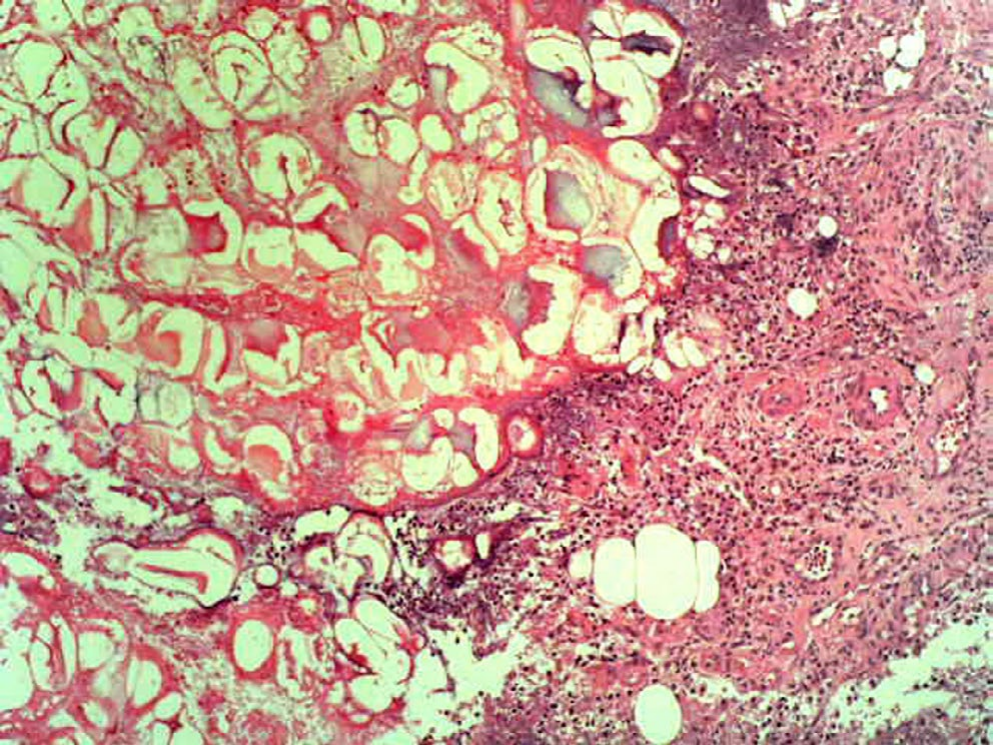
Histology of a biopsy taken from a nodus on the left leg. Again ghost‐like adipocytes are visible alongside a perivascular lymphocytic infiltrate

During the clinical course, the patient was transferred to our ward for further therapy. We saw disseminated subcutaneous dermal infiltrated, livid discolored nodules on the lower legs. The fingers were swollen and reddened with ulcers occupied by yellowish fibrinous tissue (Figure 3). Due to microbiological findings of samples taken intraoperatively from the hands and upper jaw, we escalated antibiosis with penicillin, imipenem, and amikacin. We used hydrophilic agents containing fusidic acid and betamethasone valerate for topical therapy. Serologic tests excluded an infectious disease in the form of hepatitis, HIV, or tuberculosis. A chest X‐ray did not show any evidence of sarcoidosis or tuberculosis as a possible origin of the panniculitis. Our blood samples showed an elevated lipase, leukocytosis, and mild anemia; antinuclear antibodies and antineutrophil cytoplasmic antibodies, as an indication of vasculitis, were not elevated, and a protein electrophoresis, extractable nuclear antigens screen, and immunofixation were not prominent. A combination of the elevated lipase (933 µkatal/L, standard: 1.59‐6.36 µkatal/L) with magnetic resonance imaging secured a diagnosis of a subacute pancreatitis as the cause for the lesions on the fingers and legs (Figure 4). Histology of a biopsy taken from a nodule of each leg confirmed a panniculitis showing pathognomonic ghost‐like adipocytes (Figures 5 and 6). We saw a commencing healing of the skin changes under the internal and topical therapy accompanied by the recovery of the painful joints. The patient was kept in an outpatient treatment after discharge from the hospital, and a stable condition was documented.

## 2 DISCUSSION

Considering all the clinical parameters together with imaging and serologic data, we concluded a PPP syndrome. It is a very rare combination of panniculitis and polyarthritis originating from pancreatitis. Dong et al^1^ state in a recent review that fewer than 50 cases have been published so far. According to the current literature, abdominal symptoms can often be absent.^1^ This matches our reported case, where a pancreatitis had not been diagnosed until after admission to hospital. We documented pancreatic pseudocysts in combination with pancreatic enzyme levels which led to the diagnosis of an expired pancreatitis. A pathophysiological pathway is considered where pancreatic enzymes enter the bloodstream and cause systemic fat necrosis in the subcutaneous fat tissue of the skin and in the bones and joints.^2^, ^3^ This lobular necrosis presents in the histological work‐up as ghost‐like adipocytes with lost nuclei and thickened walls due to coagulative necrosis (Figures 5 and 6).^2^, ^4^

Forms of panniculitis can be divided into septal and lobular panniculitis.^5^ An important differential diagnosis to pancreatic panniculitis is the lupus erythematodes profundus, which also causes dolent nodes on the lower extremities.^6^ It is believed to be a subset of the discoid lupus, but patients with other collagenoses, such as a systemic sclerosis, can also suffer from lupus panniculitis. A deep biopsy is recommended in all forms of panniculitis. A direct immunofluorescence is helpful to detect IgG or IgM antibodies alongside the basal membrane in order to exclude a type of Lupus. A disease that can also present nodose skin changes and arthralgia is sarcoidosis.^7^ It is a granulomatous systemic disease that typically also involves the lung with erythema nodosum on the legs. One can exclude a sarcoidosis in most cases by chest X‐ray (bilateral infiltration), electrophoresis (gamma globulin elevation), and histology (granuloma formation). Other forms of panniculitis involve calcifying panniculitis in chronic renal failure, cold panniculitis, which is mostly seen in children, or types of infectious panniculitis. Further panniculitis can be associated with rheumatoid arthritis. Necrobiosis lipoidica is described as a septal panniculitis. This disease, with a distinctive clinical appearance with yellow brown, indurated atrophic plaques most commonly in the center (unlike for our rare case) is strongly associated with diabetes. It also shows histopathological collagen degeneration, granuloma formation, fat deposition, and endothelial wall thickening.^8^ Lesions are usually painless due to associated nerve damage. Patients who do not have diabetes suffer from higher risks of getting diabetes diagnosed later in their lives. The most frequent form of panniculitis is erythema nodosum. Streptococcal infections seem to be the predominant cause, alongside other infections.^9^ Clinical manifestation is similar to other forms of panniculitis with tender subcutaneous nodules on the pretibial areas. A deep incisional biopsy with a sample of subcutaneous fat is again the option of choice for diagnosis. Depending on the stage, histology usually shows granulomatous features in early acute inflammation and fibrotic septa in late lesions.^10^ To sum up differential diagnoses, it has to be clarified that panniculitides represent a group of heterogeneous diseases involving inflammation of the subcutaneous fat. Diagnosis of a specific form of panniculitis can be difficult, as histology often shows a somewhat mixed picture where inflammatory infiltrate involves both the septa and lobules.^11^

Returning to the discussion of our case of PPP syndrome, the coagulative tissue necrosis (Figure 5) also manifests as bone necrosis, leading to a systemic polyarthritis in ankles, knees, and metacarpal joints. Further chondronecrosis is also described with involvement of articular cartilage.^12^ We report a possible involvement of necrosis in the upper jaw. As an acute pancreatitis is commonly complicated by pseudocysts in the acute inflammation, those pseudocysts are usually observed in the omental bursa and retroperitoneum. These have also recently been described to occur intrahepatically.^13^ We postulate that the radicular cyst in the maxilla could be a direct cause and presentation of the pancreatic necrosis, keeping in mind that histologic work‐up was not done in order to have a possible proof of this theory. Preliminary damage to the teeth caused by chronic nicotine consumption can further be discussed. Current knowledge of cystic lesions of the jaw is very limited. A multifactorial polygenetic pattern is discussed by Manor et al,^14^ while others mention various environmental factors (eg, viruses, bacteria, and poisons) in the formation of odontogenic cysts. A pancreatitis may also account for the acute toothache and the cyst found in the maxilla and might be another factor, as shown in this case.

## CONFLICT OF INTEREST

None declared.

## AUTHOR CONTRIBUTIONS

WK: prepared the first and final draft, implemented reviewer comments. TW: managed therapy during hospitalization and Corrected the section “Case report”. MJ: involved in corrections and markups on the whole manuscript. AA: involved in researching references and markups on the discussion. SL: served responsible for the histological findings and implementation of major revisions on the discussion.
